# Investigating a Case of Recurrent Pleural Effusion

**DOI:** 10.1155/2011/695057

**Published:** 2011-10-15

**Authors:** Patrícia Rodrigues, Maria Neves, João Pedro Ferreira, Miguel Araújo Abreu, Fernanda Almeida

**Affiliations:** Centro Hospitalar do Porto, Porto, Portugal

## Abstract

We describe the case of a patient with long-standing Parkinson's disease and recurrent bilateral pleural effusions. The pleural fluid was an exudate, rich in normal lymphocytes, and the echocardiogram, chest computerized axial tomography, and immunological, microbiological and cytological studies were negative. The patient had been taking bromocriptine, which can be related to chronic pleural effusions. Using Pubmed, we found about 40 cases of pleuropulmonary changes or constrictive pericarditis that were related to bromocriptine. We decided to suspend this drug, with resolution of the pleural effusion and respiratory complaints for more than a year now. We discuss possible underlining mechanisms for this and emphasize the importance of collecting the past medical history and medication and of considering possible iatrogenic effects.

## 1. Case Report

A 75-year-old male, with long-standing Parkinson's disease and benign prostatic hyperplasia, was brought into the emergency department after syncope. He had felt dyspnea, but denied any other prodromal symptoms and did not have involuntary movements or urinary or fecal incontinence. 

He did not have any other chronic diseases, habits, or allergies, and his medications were levodopa/carbidopa 250/25 mg qid, bromocriptine 30 mg daily, amantadine 100 mg bid, alprazolam 2.5 mg daily, and tamsulosin 0.4 mg daily.

He had been admitted to the hospital 3 times during the last 4 months with bilateral recurrent pleural effusions, dyspnea, and episodes of noncardiogenic acute pulmonary edema of unknown etiology. 

When he was admitted, he referred to dyspnea and pleuritic chest pain. The physical examination showed signs of respiratory distress, oxygen saturation was 88% in room air, and the vital signs were within normal limits, without jugular venous distension or edema. Heart sounds were normal, without murmurs, and he had no audible breath sounds on the lower half of the right lung and lower third of the left lung field, without crackles, wheezing, or rhonchi bilaterally. 

**Figure 1:**
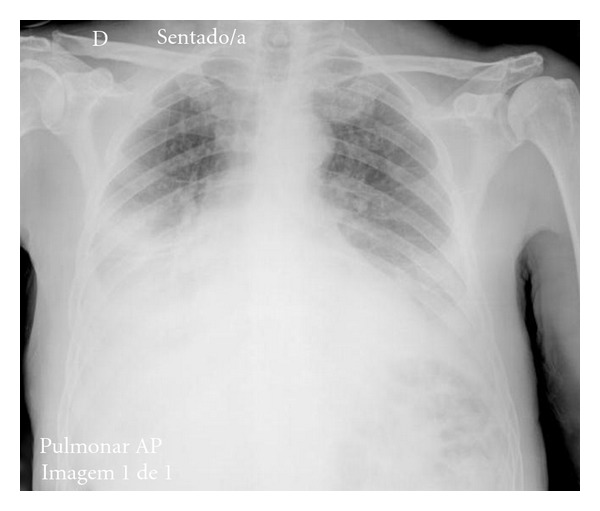
Chest X-ray on the day of admission.

The chest X-ray confirmed a big pleural effusion on the right side and a moderate one on the left (Figure 1). The pleural effusion on the right-hand side was drained and was found to be an exudate, rich in normal lymphocytes, with normal values of adenosine deaminase activity and no neoplastic cells. The pleural biopsy revealed fibrosis and moderate inflammatory infiltrate with lymphocytes, plasmocytes, and neutrophils, without granulomas, neoplastic cells, or amyloid; the bacterial cultures and polymerase chain reaction for *M. tuberculosis* were negative.

**Figure 2:**
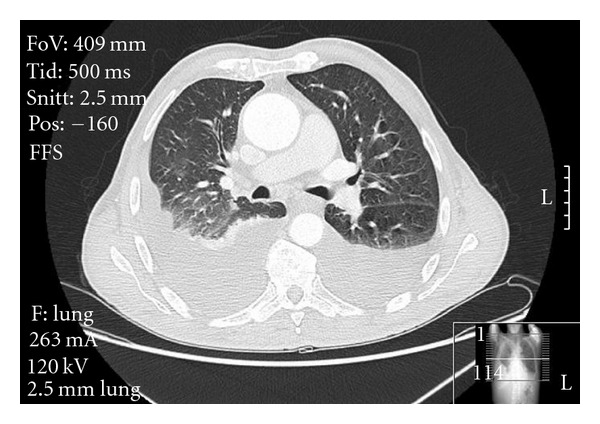
Chest computerized axial tomography shortly after the admission in the hospital.

The complete blood count, liver enzymes, creatinine, ionogram, brain (BNP natriuretic peptide), and hormone (thyroid-stimulating TSH) were normal and only the erythrocyte sedimentation rate was elevated. Blood cultures and urinalysis were negative. The PPD test (purified protein derivative) was negative (3 mm). The peripheral blood flow cytometry showed only an augmentation of NK cells, suggestive of cell activation. The transthoracic echocardiogram and EKG showed no relevant changes. The analysis of the 24-hour Holter and a 2-week electrocardiographic event recorder showed only one short episode of supraventricular tachycardia. The immunological study revealed only positive antinuclear antibodies (1/160), with immunoglobulins, complement, rheumatoid factor, antidouble stranded DNA antibodies, and antineutrophil cytoplasmic antibodies within normal values. The electroimmunoassays and detection of serum-free light chains were negative. A computerized axial tomography scan of the thorax, abdomen, and pelvis did not reveal any important changes either, with the exception of the pleural effusion and pleural thickening (Figure 2).

This case was very intriguing, because there was no apparent cause for the chronic exudative lymphocytic bilateral pleural effusions and for the episodes of pulmonary edema without heart or lung diseases.

We then decided to investigate the drugs that the patient was taking and found out that bromocriptine and other classic ergolines, such as methysergide and ergotamine, had been linked to chronic pleural effusions, generally exudates, and pleuropulmonary fibrosis. 

After failure of other treatments and drainages in avoiding recurrence of the pleural effusion, we decided to stop the bromocriptine. 

**Figure 3:**
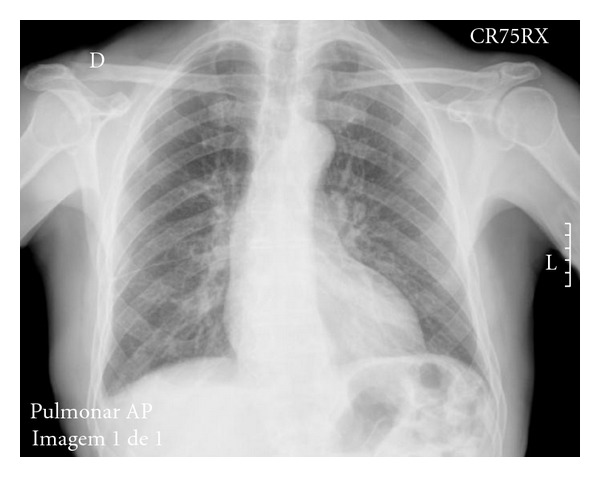
Chest X-ray one month after suspending the bromocriptine.

Within less than a month, the considerable bilateral pleural effusion disappeared (Figure 3), and the patient did not have recurrence of the pleural effusions or acute pulmonary oedema episodes since then, for more than a year now.

## 2. Discussion

Pleuropulmonary changes are rare events that have been related to bromocriptine. This association was first pointed out 30 years ago [1], but very few cases have been reported. 

In a review of the literature, using PubMed with the keywords “bromocriptine” and “pleural effusion”/“pleuropulmonary”/“pericarditis,” only about 40 cases of pleural effusions and about 4 of constrictive pericarditis were found. We did not take into account the cases where other possible confounding factors were involved, namely, asbestos exposure.

The pleural effusions related to bromocriptine are usually bilateral, exudative, rich in lymphocytes, and with fibrotic changes [2]. These changes could appear only years after starting to take the drug.

Elevated erythrocyte sedimentation rate and an inflammatory syndrome [3], as well as positive antinuclear antibodies, both seen in this patient, were also verified in other cases. Curiously, a large majority of the patients were male, over 60 years old, presenting with paroxysmal chest pain and dyspnea. Most of them had pleural thickening and pleural effusions [4–7], usually bilateral or on the right side, but some also had pulmonary interstitial infiltrates [8] and restrictive ventilatory changes [6]. Constrictive pericarditis, in some cases with associated pleural effusion, has also been described [9–11]. 

In some countries, the drug information that comes with bromocriptine already says that, in high doses and prolonged use, bromocriptine can cause pleural effusions and that, in patients presenting with pleuropulmonary signs or symptoms, the interruption of the drug should be considered, even though a clear causal effect was not found. Some brands even advise patients to do an echocardiogram, as well as a check up of the lungs, heart, and kidney function, before beginning treatment.

There are a couple of mechanisms evoked to explain these side effects: immunologic, causing a hypersensitivity response; vasoconstrictive, being a serotonin-like substance and capable of inducing fibrosis; a direct toxic fibrogenesis effect. 

However, the physiopathology of these serosal changes is not clear yet. Nevertheless, the suspension of bromocriptine was effective in this patient, as in the other described cases, and we may also speculate that the episodes of non cardiogenic pulmonary oedema could also be linked with bromocriptine pleuropulmonary changes.

Therefore, the suspension of the drug is worth trying in patients presenting with chronic pleural effusions, after excluding other causes. 

This case also reminds us of the importance of the past medical history in the diagnosis rationale and of always considering possible side effects of the medication.
